# A meta‐analysis of the effects of climate change on the mutualism between plants and arbuscular mycorrhizal fungi

**DOI:** 10.1002/ece3.8518

**Published:** 2022-01-24

**Authors:** André G. Duarte, Hafiz Maherali

**Affiliations:** ^1^ Integrative Biology University of Guelph Guelph Ontario Canada

**Keywords:** climate change, mutualism, nitrogen, phosphorus, plant biomass, root colonization

## Abstract

Climate change and other anthropogenic activities have the potential to alter the dynamics of resource exchange in the mutualistic symbiosis between plants and mycorrhizal fungi, potentially altering its stability. Arbuscular mycorrhizal (AM) fungi, which interact with most plant species, are less cold‐tolerant than other groups of fungi; warming might therefore lead to increased fungal‐mediated nutrient transfers to plants, which could strengthen the mutualism. By stimulating photosynthesis, rising CO_2_ could reduce the carbon cost of supporting AM fungi, which may also strengthen the mutualism. Furthermore, rising temperature and CO_2_ could have stronger effects on the mutualism in wild plants than in domesticated plants because the process of domestication can reduce the dependence of plants on mycorrhizal fungi. We conducted a multi‐level random effects meta‐analysis of experiments that quantified the strength of the mutualism as plant growth response to AM fungal inoculation (i.e., mycorrhizal growth response) under contrasting temperature and CO_2_ treatments that spanned the Last Glacial Maximum (LGM) to those expected with future climate change. We tested predictions using a three‐level mixed effects meta‐regression model with temperature or CO_2_, domestication status and their interaction as moderators. Increases from subambient to ambient temperature stimulated mycorrhizal growth response only for wild, but not for domesticated plant species. An increase from ambient to superambient temperature stimulated mycorrhizal growth response in both wild and domesticated plants, but the overall temperature effect was not statistically significant. By contrast, increased CO_2_ concentration, either from subambient to ambient or ambient to super ambient levels, did not affect mycorrhizal growth response in wild or domesticated plants. These results suggest the mutualism between wild plants and AM fungi was likely strengthened as temperature rose from the past to the present and that forecasted warming due to climate change may have modest positive effects on the mutualistic responses of plants to AM fungi. Mutualistic benefits obtained by plants from AM fungi may not have been altered by atmospheric CO_2_ increases from the past to the present, nor are they likely to be affected by a forecasted CO_2_ increase. This meta‐analysis also identified gaps in the literature. In particular, (i) a large majority of studies that examined temperature effects on the mutualism focus on domesticated species (>80% of all trials) and (ii) very few studies examine how rising temperature and CO_2_, or other anthropogenic effects, interact to influence the mutualism. Therefore, to predict the stability of the mycorrhizal mutualism in the Anthropocene, future work should prioritize wild plant species as study subjects and focus on identifying how climate change factors and other human activities interact to affect plant responses to AM fungi.

## Graphical abstract

We conducted a meta‐analysis of experiments that quantified plant growth response to AM fungal inoculation under contrasting temperature and CO_2_ treatments. We found that the mycorrhizal mutualism was likely strengthened by past warming due to climate change. However, mycorrhizal growth response may not have been altered by increases in atmospheric CO_2_ from the past to the present, nor will it likely to be affected by increases in CO_2_ forecasted for the future.

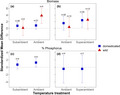

## 1 INTRODUCTION

For most of their recent evolutionary history, plants have experienced continuous changes in climatic conditions, especially atmospheric carbon dioxide (CO_2_) concentration and temperature (Franks et al., 2013; Hansen et al., 2010). At the end of the Last Glacial Maximum (LGM; approximately 20,000 years ago), CO_2_ concentrations were as low as 180 ppm (Petit et al., 1999), rising to ~410 ppm currently, and could reach between 550 and 1000 ppm by the end of the century (Ciais et al., 2013). Average global temperature also increased about 4.5°C from the LGM to the beginning of the Industrial Revolution (Otto‐Bliesner et al., 2006), and have increased an additional 0.8°C since then (Solomon et al., 2007), and may further increase between 1 and 3.7°C by the end of this century (Ciais et al., 2013; Hansen et al., 2010). Though there has been considerable focus on how plant morphology and physiology acclimate or adapt to climate change (Dusenge et al., 2018; Ehleringer et al., 2005; Temme et al., 2013), less is known about how plant interactions with other species might respond to climate change (Kiers et al., 2010). For example, plant fitness is affected by antagonists such as herbivores, and mutualists such as pollinators and soil microbes, but there is no clear consensus on how climate change modifies these effects (Bale et al., 2002; Gilman et al., 2012; Robinson et al., 2017).

One of the most widespread ecological interactions that influences plant fitness is the mutualistic symbiosis between plants and mycorrhizal fungi (Cairney, 2000; Smith & Read, 2008). In this symbiosis, plants provide fungi with up to 20% of their photosynthetically fixed carbon in exchange for increased fungal‐mediated access to soil nutrients (Jakobsen & Rosendahl, 1990; Smith & Read, 2008). Mycorrhizal fungi also benefit plants in other ways, including enhanced water uptake, protection from pathogens, and herbivore defense (Delavaux et al., 2017). The symbiosis between plants arbuscular mycorrhizal (AM) fungi is one of the most common, having evolved more than 460 million years ago (Cairney, 2000; Smith & Read, 2008) and is estimated to be present in >70% of known angiosperms (Brundrett, 2009; Maherali et al., 2016; Wang & Qiu, 2006). The symbiosis is considered a mutualism from the plant perspective because the biomass of inoculated plants is typically higher than that of non‐inoculated controls, but plant growth responses to AM fungi can span a continuum from positive to negative (Hoeksema et al., 2010; Johnson, 2010).

Variability in host plant response to AM fungi is often explained by variation in soil nutrient content (Hoeksema et al., 2010), but climate change could also influence the magnitude and direction of plant responses to AM fungi (Kivlin et al., 2013; Mohan et al., 2014). For instance, AM fungi are considered to be less cold‐tolerant than other groups of fungi (Bueno et al., 2017; Kytöviita, 2005; Maherali, 2020; Treseder et al., 2014); extraradical hyphal production and mycorrhizal colonization of roots, as well as phosphorus (P) and nitrogen (N) transfer to plants are hampered at subambient temperature (Chen et al., 2013; Liu et al., 2004; Ruotsalainen & Kytöviita, 2004), but are stimulated by warming (Bunn et al., 2009; Rillig et al., 2002). Thus, as global temperature increased from the recent geological past to present (Marcott et al., 2013; Solomon et al., 2007), the mutualistic benefit plants obtain from AM fungi may have also increased and may continue to increase with future warming (Ciais et al., 2013).

Variation in atmospheric CO_2_ concentration also has the potential to influence the mycorrhizal mutualism by altering the availability of plant‐derived carbon to the fungus. For example, growth at superambient CO_2_ increases photosynthetic rates (Drake et al., 1997; Dusenge et al., 2018) and thus the availability of carbon for AM fungi, whereas plant growth at subambient CO_2_ concentration reduces plant photosynthesis (Duarte et al., 2020; Sage & Cowling, 1999), potentially limiting the capacity of plants to provision AM fungi with carbon. In addition, by increasing carbon input from plants to the soil, rising CO_2_ can increase soil microbial activity, stimulating mineralization rates of elements such as P and N (Dhillion et al., 1996; de Graff et al., 2006), which could facilitate greater AM fungal‐mediated nutrient supply to plants. Therefore, the rise in atmospheric CO_2_ from the LGM to the present could have increased plant responsiveness to AM fungi, as well as AM fungal colonization of roots and transfers of P and N to plants. As atmospheric CO_2_ continues to rise, the intensity of the interaction between plants and AM fungi could increase (Olesniewicz & Thomas, 1999; Rillig et al., 1999; Sanders et al., 1998; Syvertsen & Graham, 1999).

Though rising temperature and CO_2_ concentration could increase the mutualistic benefit plants obtain from AM fungi, a quantitative assessment of the influence of changes in growth temperature and CO_2_ on plant responses to AM fungi is lacking. While there are meta‐analysis comparing the growth of different mycorrhizal types (i.e., arbuscular vs. ecto mycorrhizal fungi) to climate change (Dong et al., 2018; Treseder, 2004), meta‐analysis of whether climate change affects the magnitude of the mutualistic effect of mycorrhizal fungi on plant growth (i.e., the mycorrhizal growth response, Janos, 2007; Hoeksema et al., 2010), has not been done. Such an assessment is particularly important given the variability in mutualistic responses observed in the literature. For instance, mycorrhizal growth response can increase in response to experimental warming (Haugen & Smith, 1992; Smith & Roncadori, 1986), but in other cases, no effects of temperature manipulation are observed (Cabral et al., 2016; Ruotsalainen & Kytöviita, 2004; Schroeder‐Moreno et al., 2012). Similarly, mycorrhizal growth response can increase at superambient versus ambient CO_2_ in some cases (Nowak & Nowak, 2013; Zhu et al., 2016) but not others (Constable et al., 2001; Jifon et al., 2002; Zhang et al., 2015). Thus, there is no consensus on how plant response to AM fungi have changed from the LGM to the present, nor how will the mutualism change in response to future climate change.

In this study, we used a meta‐analysis to quantify how changes in temperature and CO_2_ can alter mycorrhizal growth response in plants. We quantified plant biomass and tissue % P and % N responses to AM fungal inoculation, as well as quantified changes in root colonization between plants grown at ambient conditions compared to those exposed to increased (superambient) or decreased (subambient) temperature and CO_2_ concentration, respectively. We expected that plant biomass would respond more positively to inoculation with AM fungi (i.e., a higher mycorrhizal growth response) when grown at superambient versus ambient temperature and CO_2_ concentration. By contrast, growth at subambient versus ambient temperature and CO_2_ is likely to limit the ability of AM fungi to provide nutrient acquisition services to plants, which would reduce plant responsiveness to AM fungal inoculation. Consistent with the role of AM fungi as nutritional symbionts, we also expected that % P and % N in plant tissues would be higher in inoculated versus non‐inoculated controls for plants grown at higher temperature and CO_2_. If AM fungal growth is stimulated at higher temperature and CO_2_ concentration, then we also expected that the magnitude of AM fungal colonization in inoculated plants would increase with temperature and CO_2_ concentration. Because artificial selection to maximize productivity in fertilized fields during the process of domestication can reduce the mycorrhizal responsiveness of plants to AM fungi (Frederickson, 2017; Martin‐Robles et al., 2018; Tawaraya, 2003), we quantified effect sizes for domesticated and wild plants separately. We expected that mycorrhizal growth response would be more strongly influenced by temperature and CO_2_ variation in wild plants than in domesticated plants.

## 2 MATERIALS AND METHODS

### 2.1 Data collection

To assess whether the magnitude of plant response to AM fungi varied with temperature and CO_2_, we surveyed the literature for studies that reported biomass for plants inoculated with AM fungi and for corresponding non‐inoculated control. The literature was searched using Google Scholar and Web of Science with the keywords: “mycorrhiza and temperature,” “mycorrhiza and heat,” “mycorrhiza and cold,” “mycorrhiza and CO_2’_” and “mycorrhiza and carbon dioxide.” We structured our search following the recommendations from the preferred reporting items for systematic reviews and meta‐analysis (O’Dea et al., 2021). We searched for any article that had both search keywords in any part of text, which resulted in more than 150,000 results across all search strings. We screened 50–100 pages of titles in each search engine (10 titles/page) for each search term to identify studies that were potentially relevant. Based on the title, we identified 1013 studies that could potentially have the information of interest. A screen of the abstracts showed that 316 articles might contain the required information, and a full‐text review of these articles identified 62 that contained all the necessary information to be included in the meta‐analysis. Ten articles were excluded from this list due to experimental design issues or a lack of information on variance and sample size (Figure S1; Table S1).

To be included in the meta‐analysis, experiments must have been done with plants grown at both ambient temperature or CO_2_ concentration as well as at temperature or CO_2_ below and/or above ambient conditions. We included both chamber and field studies that exposed plants to temperature or CO_2_ treatments since germination, that is, plants that had been germinated or transplanted upon germination to the treatment conditions. If a study included more than one plant or mycorrhizal species, each combination of plant and fungal species was counted as separate trial. In addition, the combinations of unique plant and AM fungal ecotypes or genotypes, as identified by study authors, even if they belonged to the same species pairs, were also considered separate trials. In experiments that manipulated fertilizer levels, we selected the treatment that the study identified as the control or the treatment where soil mineral nutrients had not been manipulated. In cases where the variables had been measured at more than one time point, the last sampling date was used, as that represented the longest stage to which the plants have been exposed to the treatments. In all the selected studies, we also collected data on % P and % N of plant tissue and percent root colonization by AM fungi, if available. For % P and % N, studies reported either whole plant values, the sum of root and shoot values, or shoot or root values only. We collected nutrient and colonization data only from the studies that also reported biomass so that these variables could be compared using the same pool of studies.

Plant species were classified as either domesticated or wild based on the information provided by the authors in each paper. A plant species was considered domesticated if it had a history of human‐directed breeding either by methodical or unconscious selection (Darwin, 1868; Ross‐Ibarra et al., 2007). Thus, species identified as crops or cultivars were considered to be domesticated. Plant species collected from wild populations or lacking a history of human‐mediated selection were designated as wild species.

The majority of trials involved manipulation of temperature or CO_2_ treatments between subambient and ambient levels or between ambient and superambient levels. A minority of trials included all three levels of the temperature or CO_2_ treatments (Table S1). We found that 20 papers on temperature and 32 papers on CO_2_ treatments contained the necessary information for the analysis (Table S1). For temperature studies, 12 manipulated air temperature and 8 manipulated soil temperature. The analysis of temperature effects resulted in 151 trials and included 22 plant species (13 domesticated and 9 wild species) and 14 AM fungal species. The analysis of CO_2_ effects resulted in 115 trials and included 45 plant species (20 domesticated and 25 wild species) and 7 AM fungal species. A majority of studies reported plant growth as total dry biomass (including the sum of shoots and roots), but there were a few cases where only a component of biomass was reported. For studies manipulating temperature, one reported only root biomass, three reported only shoot biomass, and one reported only bulb biomass. Removing those studies from the meta‐analysis did not change the results, so they were included in the analysis. For studies that manipulated CO_2_, total biomass data were available, or both shoot and root biomasses were reported, so that total biomass could be calculated from their sum.

Data extraction consisted of obtaining the mean, standard deviation (SD), and sample size of the subambient, ambient, and superambient temperature or CO_2_ treatments for both inoculated and non‐inoculated control plants. Treatments were classified as subambient, ambient, or superambient according to how they were identified in the original study (e.g., superambient for treatments that were artificially warmed or received supplemental CO_2_, and ambient for those not manipulated). Data were extracted from main text, tables, supplemental material, or from plots using the software DataThief (B. Tummers, DataThief III. 2006, <https://datathief.org/>). In cases where SD was not given, it was calculated from the *P*‐value, according to Higgins and Green (2008). In such cases, we used the *P*‐value and degrees of freedom to obtain the corresponding *t*‐value. To calculate the standard error, the difference in the mean of the groups being compared was divided by the *t*‐value. The SD was then calculated by dividing the standard error by the inverse square root of the sample size.

### 2.2 Statistical analysis

Mycorrhizal growth response is known to vary among studies because of the differences in growth conditions, particularly nutrient supply and the identity of plant and AM fungal species (Hoeksema et al., 2010). Consequently, comparisons of mycorrhizal growth response across different temperature, CO_2_ or environmental treatments should be done using the same population of studies (Maherali, 2014). To ensure that this condition was met, the trials were grouped into two categories that reflected design of the original studies, that is, those that (i) compared mycorrhizal response at subambient and ambient temperature or CO_2_ and (ii) compared mycorrhizal response at ambient and superambient temperature or CO_2_. A minority of studies contained subambient, ambient, and superambient temperature or CO_2_ treatments. The data in these latter studies were subdivided to fit into the first two categories described above.

A multi‐level random effects meta‐analysis (Harrer et al., 2021) was used to quantify the responses of plant biomass and tissue % P and % N to AM fungal inoculation in each treatment combination of temperature, CO_2_ or plant domestication status subgroups. The response of plant biomass and tissue % P and % N to AM fungal inoculation was calculated as the standardized mean difference (SMD) between inoculated treatments and non‐inoculated controls, which is equivalent to Hedges’ g (Harrer et al., 2021). This metric is analogous to the mycorrhizal growth response where positive values indicate that inoculated plants are larger than non‐inoculated plants and negative values indicate the opposite (Hoeksema et al., 2010). All analysis were performed in R 4.2.0 using the packages “meta” and “dmetar” (Harrer et al., 2021).

The multi‐level random effects meta‐analysis included three nested levels to account for (i) variation in plant and fungal species identities among trials included within a study, (ii) variation among studies in experimental conditions such as length of experiment, location, greenhouse characteristics, and soil nutrients, and (iii) variation among studies within temperature, CO_2,_ or domestication status subgroups (Harrer et al., 2021). Effect sizes were calculated by weighting each trial based on the inverse of its variance (Borenstein et al., 2009) and 95% confidence intervals were used to determine if the summary effect was significantly different from zero (i.e., an effect was considered statistically significant when the confidence interval did not overlap zero).

To test for the effects of temperature or CO_2_ concentration, domestication status and their interaction on the response of biomass, % P and % N to AM fungal inoculation, we used a three‐level mixed effects meta‐regression model with temperature or CO_2_, domestication status, and their interaction as moderators (Harrer et al., 2021). Because of the unbalanced design, restricted maximum likelihood (REML) was used to calculate variance components and determine statistical significance of the moderators and their interaction (Borenstein et al., 2009).

We also used a three‐level random effects meta‐analysis to evaluate whether % AM fungal colonization of roots was affected by temperature and CO_2_. We calculated the SMD as the difference between ambient versus the subambient temperature or CO_2_ treatment and as the difference between the superambient and ambient temperature or CO_2_ treatment for domesticated and wild species. As a result, if % AM fungal colonization of roots increased in response to a rise in temperature or CO_2_, the SMD values would be positive. To determine whether plant domestication status influenced the response of AM fungal colonization to temperature or CO_2_, we used a three‐level mixed effects meta‐regression model with domestication status as the moderator (Harrer et al., 2021). As with the meta‐regression described in the previous paragraph, REML was used to calculate variance components and determine whether the moderator had a statistically significant effect.

To determine whether publication bias was present, effect sizes were plotted against standard errors in funnel plots, and the asymmetry of these plots was calculated with an Egger's test of the intercept (Egger et al., 1997).

## 3 RESULTS

The average and range of temperature and CO_2_ concentration used in the studies included in the meta‐analysis are shown in Table 1. In studies that compared subambient and ambient temperature, mean subambient temperature was about 8.3°C cooler than the ambient treatment. In studies that compared ambient and superambient temperature, the superambient treatment was in average 9.5°C warmer than the ambient treatment. In studies that compared subambient and ambient CO_2_ treatments, mean CO_2_ concentration was ~188 ppm lower in the subambient compared to the ambient treatment. In studies that compared ambient to superambient CO_2_, mean [CO_2_] was ~345 ppm higher in the superambient than in the ambient treatment. For both temperature and CO_2_, the ambient treatments had a narrower range of values compared to the sub‐ and superambient treatments.

**TABLE 1:** Mean (± SD) and range and of temperature and CO_2_ treatments imposed by the studies included in the meta‐analysis

Treatment comparison	Mean	Range
Temperature		
Subambient to ambient studies (13)		
Subambient	17.28 ± 3.40°C	8–24°C
Ambient	25.63 ± 2.68°C	12–30°C
Ambient to superambient studies (14)		
Ambient	21.68 ± 5.43°C	12–26°C
Superambient	31.17 ± 6.42°C	17–42°C
CO_2_		
Subambient to ambient studies (3)		
Subambient	207.14 ± 65.25 ppm	100–270 ppm
Ambient	395 ± 5.77 ppm	390–400 ppm
Ambient to superambient studies (30)		
Ambient	398.98 ± 37.71 ppm	300–450 ppm
Superambient	743.68 ± 153.96 ppm	550–1500 ppm

Note

The numbers in parenthesis represent the number of studies in each treatment

**FIGURE 1:**
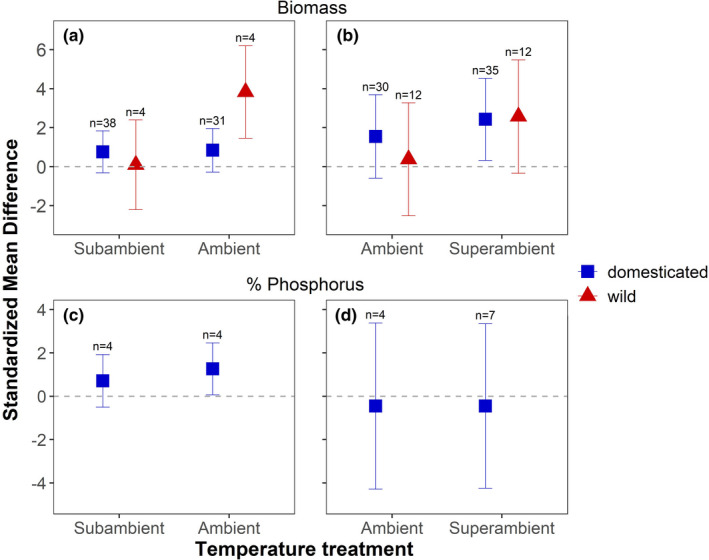
The mycorrhizal growth response of wild and domesticated plants to AM fungal inoculation in studies that manipulated subambient and ambient temperature (a), and ambient and superambient temperature (b). The response of % P in plant tissues of domesticated plants to AM fungal inoculation in studies that manipulated subambient and ambient temperature (c), and ambient and superambient temperature (d). Symbols represent the standardized mean difference (SMD) and error bars represent the 95% confidence intervals. Whether an SMD was significantly different from zero was determined by whether the error bars overlapped zero. The sample size (*n*) is the number of trials at each temperature treatment and domestication status subgroup

In studies that compared subambient and ambient temperature, mycorrhizal growth response, calculated as the standardized mean difference (SMD) of plant biomass in inoculated treatments versus non‐inoculated controls, was affected by domestication status (*p* = .03) and the interaction between domestication status and temperature treatment (*p* < .01) (Table 2). Specifically, the mycorrhizal growth response of domesticated species did not vary with temperature (subambient SMD = 0.76; ambient SMD = 0.84; Figure 1a). By contrast, the mycorrhizal growth response of wild species increased substantially from subambient (SMD = 0.09) to ambient temperature (SMD = 3.83).

**TABLE 2:** Results from the three‐level mixed‐effects meta‐regression analysis testing the effect of temperature treatment, domestication status (domesticated or wild) and their interaction on biomass, tissue % P, and % AM fungal colonization of roots

Factor	Subambient to ambient temperature studies						Ambient to superambient temperature studies					
	Biomass		% P		% Colonization		Biomass		% P		% Colonization	
	*t*	*p*	*t*	*p*	*t*	*p*	*t*	*p*	*t*	*p*	*t*	*p*
Temperature	−.181	.857	1.431	.202			1.483	.142	.800	.994		
Domestication status	2.268	**.026**			−1.425	.169	−.644	.521	.214	.835	1.139	.265
Temperature × Status	−2.735	**.008**					1.168	.246	1.359	.201		

Note

The *t*‐value and *p*‐value (bolded when significant at the 0.05 level) are shown for each moderator and the interaction of the two moderators, when applicable.

In studies that compared ambient and superambient temperature (Figure 1b), mycorrhizal growth response was not affected by either temperature, domestication status or the interaction of the two moderators (Table 2). Though the temperature effect was not significant in the overall model, we note that mycorrhizal growth response increased in domesticated species from ambient (SMD = 1.55) to superambient (SMD = 2.43) temperature, with the latter value being significantly greater than zero. There was also a marked increase in the mycorrhizal growth response of wild species from ambient (SMD = 0.38) to superambient (SMD = 2.57) temperature, but neither value was significantly different from zero.

The response of plant % P to AM fungal inoculation did not differ across temperature treatments, neither between comparisons of subambient and ambient temperature (Figure 1c), nor between ambient and superambient temperature (Figure 1d) (Table 2). In studies that compared subambient and ambient temperature, the response of % P in domesticated plants was significantly >0 at ambient temperature (SMD = 1.26) but did not differ from zero at subambient temperature (SMD = 0.71). In studies that compared ambient and superambient temperature, the response of plant % P was not different from zero at ambient or superambient temperature in domesticated plants. Not enough data were available in the published literature to calculate the response of % P to AM fungi at different temperatures in wild species or the response of % N to AM fungi at different temperatures in either wild or domesticated species.

**FIGURE 2:**
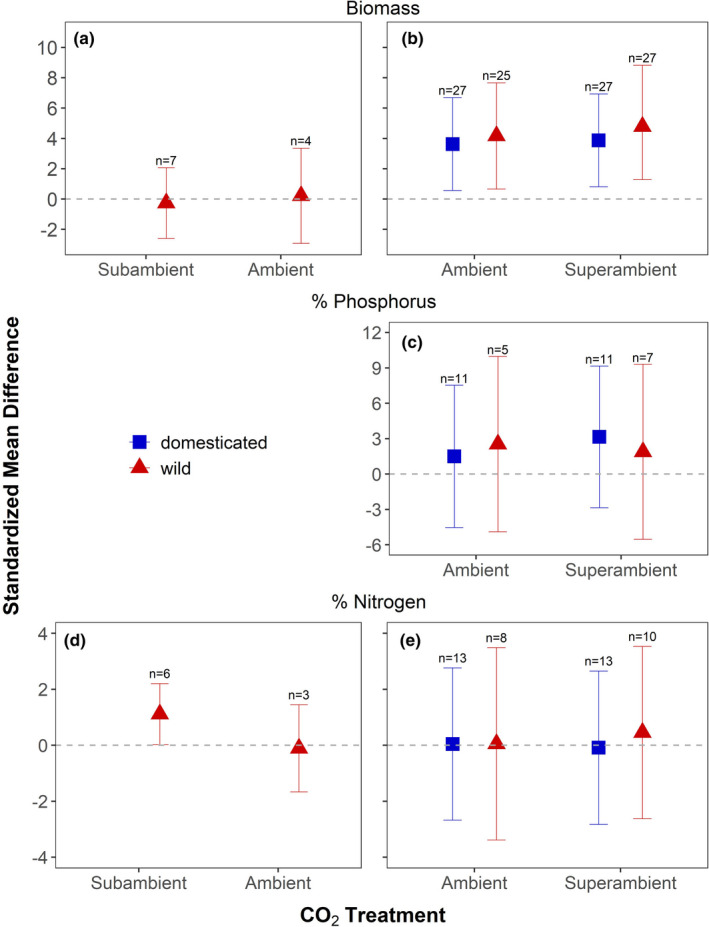
The mycorrhizal growth response of wild and domesticated plants to AM fungal inoculation in studies that manipulated subambient and ambient CO_2_ (a), and ambient and superambient CO_2_ (b). The response of % P in plant tissues of wild and domesticated plants to AM fungal inoculation in studies that manipulated ambient and superambient CO_2_ (c). The response of % N in plant tissues of wild and domesticated plants to AM fungal inoculation in studies that manipulated subambient and ambient CO_2_ (d) and ambient and superambient CO_2_ (e). Symbols represent the standardized mean difference (SMD) and error bars represent the 95% confidence intervals. Whether an SMD was significantly different from zero was determined by whether the error bars overlapped zero. The sample size (*n*) is the number of trials at each CO_2_ treatment and domestication status subgroup

In studies that compared subambient and ambient CO_2_ in wild species, mycorrhizal growth response did not differ across CO_2_ concentration (Table 3) and was not different from zero in either treatment (SMD = −0.26 and 0.22, respectively; Figure 2a). We note that there were too few studies of domesticated species to permit a comparison of mycorrhizal growth response between subambient and ambient CO_2_. In studies that compared ambient and superambient CO_2_, mycorrhizal growth response was significantly greater than zero for both wild (SMD = 4.17 and 4.78 at ambient and superambient CO_2_, respectively) and domesticated plants (SMD = 3.62 and 3.87 at ambient and superambient CO_2_, respectively) (Figure 2b). Nonetheless, neither the effects of CO_2_ concentration, domestication status, nor their interaction influenced mycorrhizal growth response (Table 3).

**TABLE 3:** Results from the three‐level mixed‐effects meta‐regression analysis testing the effect of CO_2_ treatment, domestication status (domesticated or wild), and their interaction on biomass, tissue % P, tissue % N, and % AM fungal colonization of roots

Factor	Subambient to ambient CO_2_ studies						Ambient to superambient CO_2_ studies							
	Biomass		% N		% Colonization		Biomass		% P		% N		% Colonization	
	*t*	*p*	*t*	*p*	*t*	*p*	*t*	*p*	*t*	*p*	*t*	*p*	*t*	*p*
CO_2_	−.279	.786	1.520	.172			.463	.644	1.193	.242	−.068	.946		
Domestication status					1.449	.162	.404	.687	.331	.743	.004	.997	.101	.920
CO_2_ × Domestication status							.470	.639	−1.011	.320	.179	.859		

Note

The *t*‐value and *p*‐value (bolded when significant at the 0.05 level) are shown for each moderator and the interaction of the two moderators, when applicable.

The response of plant % P to AM fungal inoculation in studies that compared ambient and superambient CO_2_ was not affected by CO_2_ concentration nor domestication status, nor their interaction (Table 3). Though the response to AM fungal inoculation was positive in all cases, SMDs were not significantly different from zero (Figure 2c). The response of plant % P to AM fungi at subambient and ambient CO_2_ could not be calculated in either wild or domesticated studies due to a lack of published studies.

Only data on wild species were available to calculate the response of % N to AM fungal inoculation in studies that compared subambient and ambient CO_2_ (Figure 2d). The response of plant % N to AM fungal inoculation was not affected by CO_2_ treatment (Table 3) but was significantly greater than zero at subambient CO_2_ (SMD = 1.11) and not different from zero at ambient CO_2_ (SMD = −0.11). In studies that compared ambient and superambient CO_2_ (Figure 2e), the response of % N to AM fungal inoculation was not affected by CO_2_ concentration nor domestication status, nor their interaction (Table 3). In addition, the response of % N to AM fungi was not significantly different from zero for any treatment combination of domestication status and CO_2_ concentration.

**FIGURE 3:**
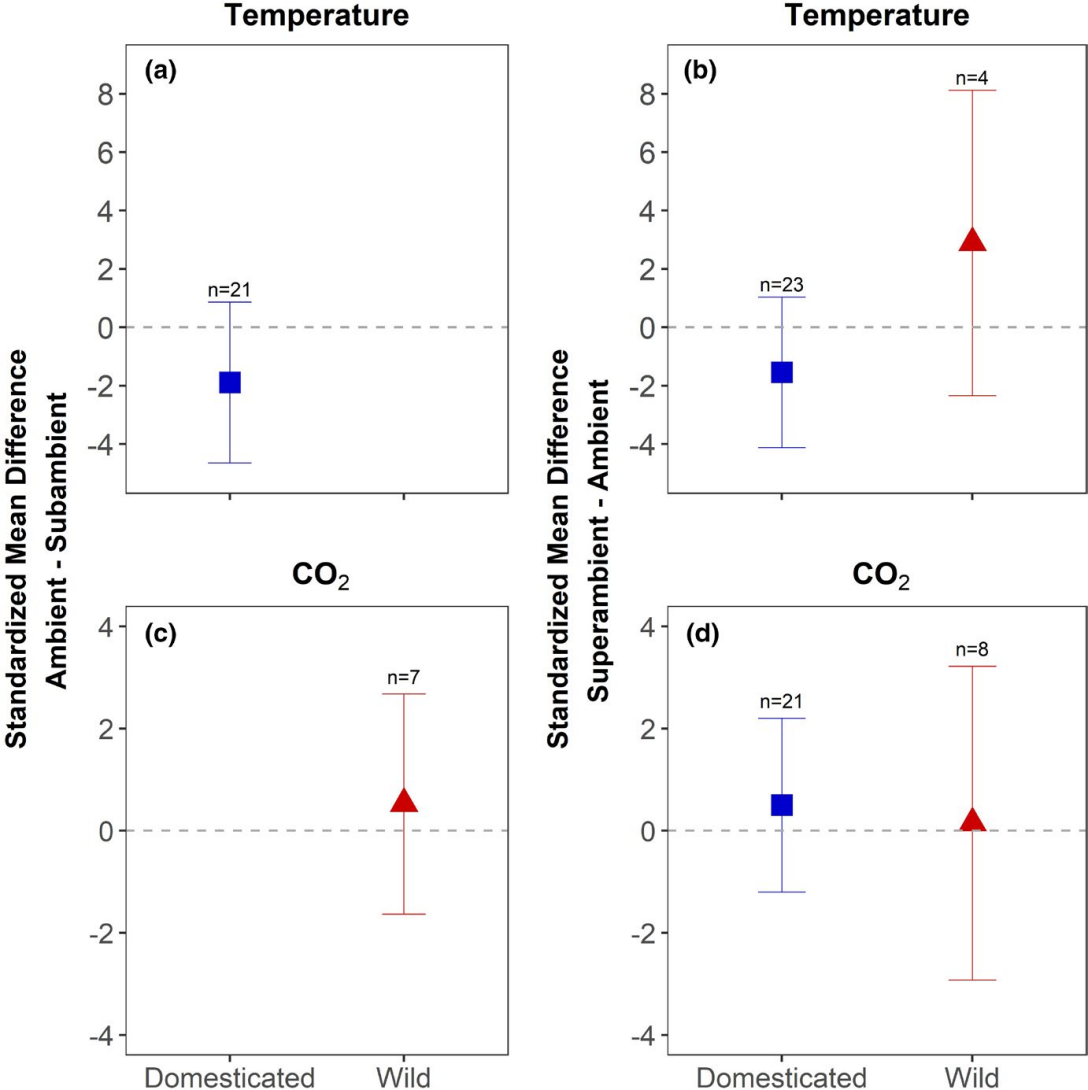
The response of % AM fungal colonization of roots to subambient temperature in relation to ambient temperature (a), and superambient temperature in relation to ambient temperature (b) in domesticated and wild plants. The response of % AM fungal colonization of roots to subambient CO_2_ in relation to ambient CO_2_ (c), and superambient CO_2_ in relation to ambient CO_2_ (d) in domesticated and wild plants. Circles represent the mean standard difference (SMD), and error bars represent the 95% confidence intervals. Whether an SMD was significantly different from zero was determined by whether the error bars overlapped zero. Because the SMD is calculated as the difference between ambient versus the subambient temperature or CO_2_ treatment and as the difference between the superambient and ambient temperature or CO_2_ treatment, a positive value means that % AM fungal colonization of roots increased in response to a rise in temperature or CO_2_. The sample size (*n*) is the number of trials in each domestication status subgroup

The colonization of roots by AM fungi in inoculated plants was not affected by changes in growth temperature, either for comparisons between subambient and ambient treatments or comparisons between ambient and superambient treatments (Table 3; Figure 3a,b). We note that the response of AM fungal colonization to a change from subambient and ambient temperature could only be calculated for domesticated species; there were insufficient published data for wild species (Figure 3a). The colonization of roots by AM fungi was also unaffected by changes in CO_2_ concentration, either for comparisons between subambient and ambient treatments or between ambient to superambient treatments (Table 3, Figure 3c,d). The response of AM fungal colonization to a change from subambient to ambient CO_2_ could only be calculated for wild species; there were insufficient published data for domesticated species.

Publication bias, assessed with Funnel plots and Egger's test of the intercept (Egger et al., 1997), was observed in 32% of subgroups (Figure S2). When significant publication bias was detected, it was caused by trials that had low weight due to high variances (and thus small sample sizes) and as a result did not have a meaningful effect on the overall effect size for subgroups (Bettoni et al., 2014; Humphreys et al., 2010; Smith & Roncadori, 1986). In addition, the primary focus of the meta‐analysis was on the effect of temperature, CO_2,_ and their interaction on mycorrhizal growth response, not the summary effect size of mycorrhizal growth response in each subgroup. To facilitate statistical tests of the effect of moderators on mycorrhizal growth response, no studies were removed from the three‐level mixed effects meta‐regression model.

## 4 DISCUSSION

The results of this meta‐analysis suggest that climate change factors differ in their effects on the mutualistic benefits plants obtain from AM fungi. Furthermore, the effects of climate change on plant responses to AM fungi, when detected, can differ between wild and domesticated plants. For example, we found that temperature increases from subambient to ambient levels stimulated mycorrhizal growth response in wild species, but not domesticated species (Figure 1a). Though not statistically significant, we also observed an increase in the summary effects size for mycorrhizal growth response in both domesticated and wild species from ambient to superambient temperature. By contrast, manipulating CO_2_ concentration did not affect mycorrhizal growth response, neither from subambient to ambient CO_2_ nor from ambient to superambient CO_2_. Therefore, the rise in temperature from the past to the present could have strengthened the mutualistic effects of AM fungi on plants, whereas projected temperature increases may have weaker, though still positive, effects on the growth benefits plants obtain from AM fungi. Mycorrhizal growth response may neither have been appreciably altered by increases in atmospheric CO_2_ from the past to the present, nor will it likely be affected by increases in CO_2_ forecasted for the future.

As expected, mycorrhizal growth response in wild plants was more strongly influenced by temperature than in domesticated plants. Specifically, mycorrhizal growth response increased substantially from subambient to ambient temperature in wild plants, whereas no change was observed in domesticated plants (Figure 1a). The effect of temperature was not statistically significant in the mixed effects meta‐regression model for studies that compared ambient and superambient temperature; however, the summary effect size increased by 2.19 units in response to warming for wild species, which was more than double the increase of 0.88 units observed for domesticated species (Figure 1b). Thus, a history of artificial selection during the process of domestication may have decreased the temperature sensitivity of the mycorrhizal mutualism in domesticated plants. We note that far fewer studies have evaluated temperature effects on mycorrhizal growth response in wild than domesticated plants (29/151 trials, or 19%; Table S1). Consequently, estimates of temperature effects on the mycorrhizal growth response in wild plants, and conclusions about differences in temperature sensitivity of the mycorrhizal mutualism between wild and domesticated plants should be considered preliminary.

We expected mycorrhizal growth response would increase with growth temperature because AM fungi are considered less cold tolerant than other groups of fungi, and warmer temperature should increase AM fungal supply of P and N to plants (Bunn et al., 2009; Chen et al., 2013; Gavito et al., 2003; Liu et al., 2004; Rillig et al., 2002; Ruotsalainen & Kytöviita, 2004). Though the mutualistic effect of AM fungi on plant growth increased from subambient to ambient temperature and from ambient to super ambient temperature, particularly for wild plants, we did not detect a positive effect of temperature on the response of plant tissue % P to AM fungal inoculation (Figure 1c,d). As a result, enhanced mycorrhizal‐mediated nutrient uptake with increased temperature may not necessarily be the mechanism responsible for increased mycorrhizal growth response. However, we note that of the 151 trials examining mycorrhizal growth response to temperature, only 29 measured % P (Table S1) and there were an insufficient number of studies to calculate the response of % N to AM fungal colonization among subgroups. Furthermore, nutrient concentrations were measured primarily in studies on domesticated plants. This limited sample size was likely too small to detect temperature effects on AM fungal‐mediated nutrient uptake. Identifying the magnitude of temperature effects on mycorrhizal growth response and their underlying mechanisms should be priorities for future research.

One caveat to predicting how climate change influences mycorrhizal growth response is that temperature manipulations done in experiments do not necessarily match the magnitude of climate change from the recent past to the present or that expected for future climate change. For example, subambient temperature manipulations averaged across studies were 8.3°C below ambient (Table 1), whereas the change in temperature from the LGM to the present day was a more modest ~5°C increase (Otto‐Bliesner et al., 2006; Solomon et al., 2007). Therefore, it is possible that the larger magnitude of temperature manipulations in experimental studies overestimate the temperature sensitivity of mycorrhizal growth response. However, it should be noted that some estimates place average global surface temperature during the LGM as low as 9°C (Otto‐Bliesner et al., 2006; Petit et al., 1999), which was much colder than the average subambient temperature in studies that contributed to the meta‐analysis (~17°C). If temperatures <17°C further suppress the mutualistic quality of AM fungi (Kytöviita, 2005), then plant responses to AM fungi during the LGM may have been lower than that estimated by the SMD calculated at subambient temperature (Figure 1a).

For studies that compared ambient and superambient temperature, the average temperature treatment increase of ~9.5°C (Table 1) was far in excess of a globally averaged expected warming of between 1 and 3.7°C by the end of this century (Ciais et al., 2013; Hansen et al., 2010). The higher magnitude of warming in experimental studies could have exceeded the temperature optima for photosynthesis for many C_3_ species, as well as increased respiration (Way & Yamori, 2014). These temperature‐dependent declines in net carbon acquisition could have increased the carbon cost of the mycorrhizal mutualism for plants (Fellbaum et al., 2012; Smith et al., 2009). If increased carbon costs due to excessive warming in experiments offsets the benefit of potentially greater AM fungal‐mediated acquisition of P and N, then the relatively modest effect of an increase in temperature from ambient to superambient levels on mycorrhizal growth response we observed (Figure 1b) may underestimate what would occur in response to the globally averaged temperature increase predicted by climate change models.

We predicted that because increased CO_2_ concentration stimulates photosynthesis (Drake et al., 1997; Dusenge et al., 2018) and reduces the relative cost of supplying carbon to AM fungi, mycorrhizal growth response would increase with treatment CO_2_ concentration (Gavito et al., 2003). In contrast to expectations, there was no effect of increased CO_2_ concentration on mycorrhizal growth response (Figure 2a,b). This result was similar for both wild and domesticated species in studies that compared ambient and superambient CO_2_, suggesting that future increases in atmospheric CO_2_ will not affect the relative benefits and costs of the AM fungal mutualism for plants (Gavito et al., 2003; Terrer et al., 2016). Assessing how mycorrhizal growth response was affected by CO_2_ increases from the LGM to the present was limited by low sample size; very few studies have investigated mycorrhizal growth response at subambient CO_2_ (7/115 trials, or 6%; Figure 2a). Though limited, prior work also shows that mycorrhizal growth response can be stimulated by subambient CO_2_ in some species but suppressed in others (Becklin et al., 2016). Given this variability and small sample size, additional research is needed to determine if the mycorrhizal response of both wild and domesticated species have been affected by the rise in CO_2_ concentration from the LGM to the present.

Because AM fungal growth should be more limited at colder temperature and negatively affected by carbon limitation, we predicted that subambient temperature and CO_2_ would inhibit AM fungal colonization of roots relative to ambient conditions, whereas the opposite would be observed for plants grown at superambient versus ambient temperature and CO_2_ (Liu et al., 2004; Olesniewicz & Thomas, 1999; Rillig et al., 1999; Ruotsalainen & Kytöviita, 2004; Sanders et al., 1998; Syvertsen & Graham, 1999). These predictions were not supported; AM fungal colonization of roots did not differ between temperature (Figure 3a,b) or CO_2_ treatments (Figure 3c,d). Nonetheless, we note that trials examining root colonization responses to temperature and CO_2_ were dominated by domesticated plants (42/46 trials for temperature [91%] and 21/34 trials for CO_2_ [62%]). Consequently, the conclusion that root colonization is insensitive to the manipulation of temperature and CO_2_ is more robust for domesticated species.

This meta‐analysis highlights additional priorities for future research. In particular, multiple climate change factors are expected to have interactive effects on plant functioning (Norby & Luo, 2004), including responses to the mycorrhizal mutualism (Kivlin et al., 2013; Mohan et al., 2014). For instance, elevated CO_2_, by stimulating photosynthesis, could further enhance the effects of warming on mycorrhizal growth response. However, only a limited number of studies have tested this prediction (Büscher et al., 2012; Gavito et al., 2003). Another important priority is to explore how climate change will interact with other human effects on the environment, particularly increased nutrient deposition. Increased N and P deposition (Bouwman et al., 2017; Peñuelas et al., 2013) could potentially counteract the positive effects of warming on plant response to AM fungi because plants would be less likely to benefit from AM fungal mediated nutrient acquisition services (Johnson, 2010). Only a small number of studies have explored how climate change factors interact with nutrient deposition to affect the magnitude and direction of plant response to AM fungi and a consensus about the magnitude and direction of these interactive effects has not emerged (Jakobsen et al., 2016; Kivlin et al., 2013; Syvertsen & Graham, 1999; Watts‐Williams et al., 2019). Therefore, though this meta‐analysis indicates that climate change factors acting in isolation are not likely to cause the mycorrhizal mutualism to break down (Frederickson, 2017), research on how climate change factors and other human activities interact to affect plant responses to AM fungi is needed to predict the stability of the mycorrhizal mutualism in the Anthropocene.

## CONFLICT OF INTEREST

The authors declare there are no conflicts of interest.

## AUTHOR CONTRIBUTIONS

**André G. Duarte:** Conceptualization (equal); Data curation (equal); Formal analysis (equal); Investigation (equal); Methodology (equal); Validation (equal); Visualization (equal); Writing – original draft (equal); Writing – review & editing (equal). **Hafiz Maherali:** Conceptualization (equal); Data curation (equal); Formal analysis (equal); Funding acquisition (equal); Investigation (equal); Methodology (equal); Project administration (equal); Supervision (equal); Validation (equal); Visualization (equal); Writing – original draft (equal); Writing – review & editing (equal).

## Supporting information

Fig S1

Fig S2

Table S1

## Data Availability

The data that supports the findings of this study are available in the supplementary material of this article.
