# Supplementary material for: A meta‐analysis of the effects of climate change on the mutualism between plants and arbuscular mycorrhizal fungi
Source: Ecol Evol. 2022 Jan 24;12(1):e8518. doi: 10.1002/ece3.8518 (PMC8796888; doi:10.1002/ece3.8518)

Figure S1. Recorded entries from the literature survey following the structure of the preferred reporting items for systematic reviews and meta-analysis (PRISMA).

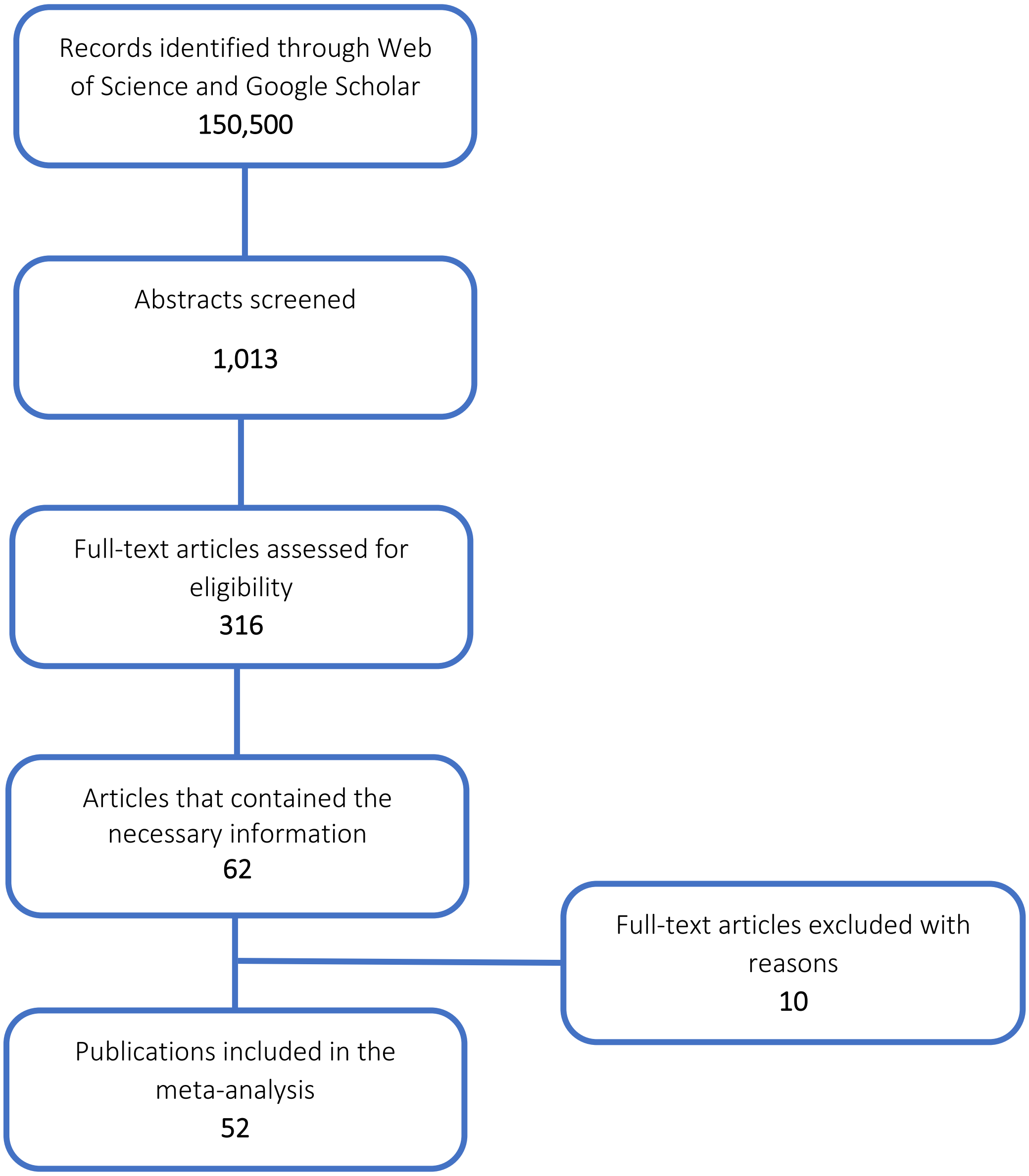

Supplement: Supplementary file 1 — Fig S1 [file ECE3-12-e8518-s001.pdf]
