# Supplementary material for: A meta‐analysis of the effects of climate change on the mutualism between plants and arbuscular mycorrhizal fungi
Source: Ecol Evol. 2022 Jan 24;12(1):e8518. doi: 10.1002/ece3.8518 (PMC8796888; doi:10.1002/ece3.8518)

Figure S2. Funnel plots testing for publication bias for each dependent variable, grouped by temperature and CO_2 treatments and domestication status. The asymmetry of the plots was tested using the Egger's test of the intercept, considering a *p*-value as significant when ≤ 0.05. The numbers on the plots correspond to each trial as listed in Table S1.

Temperature

Biomass

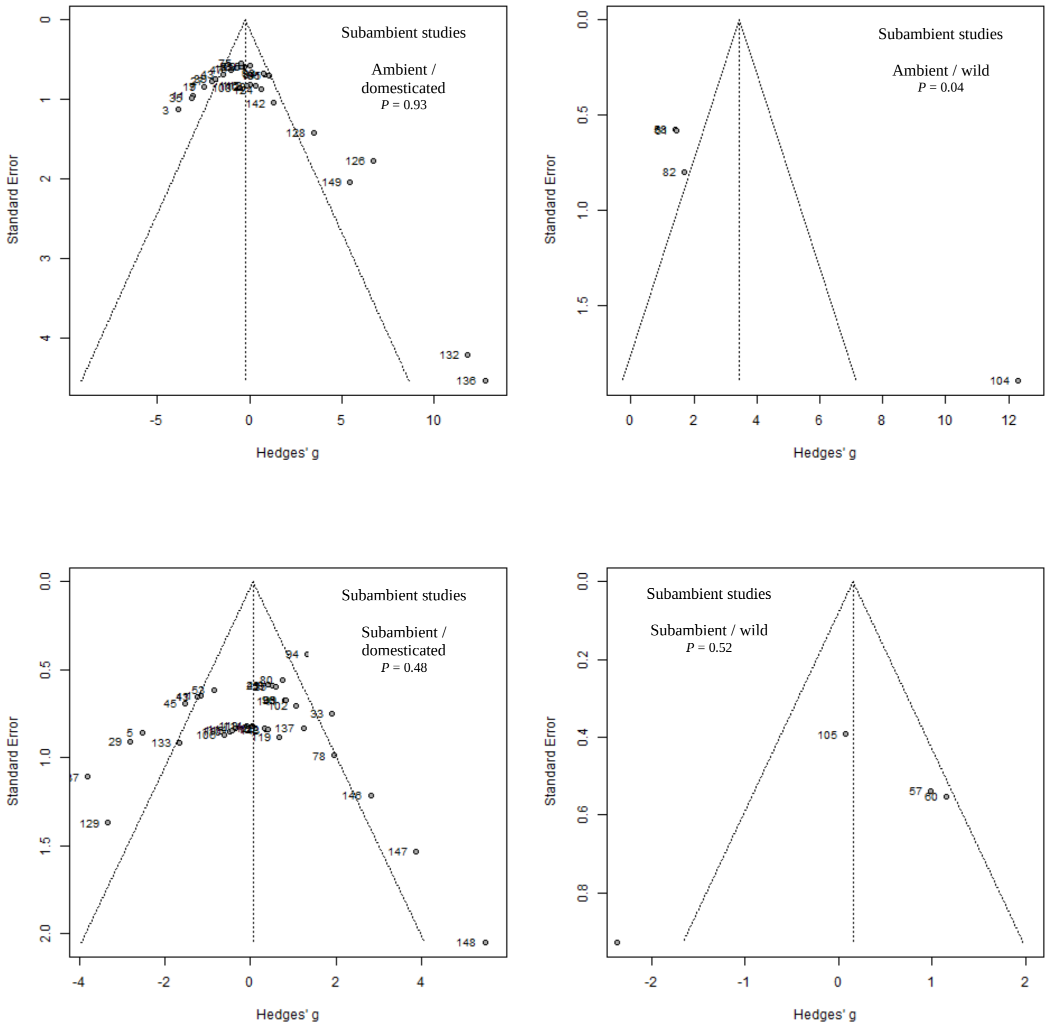

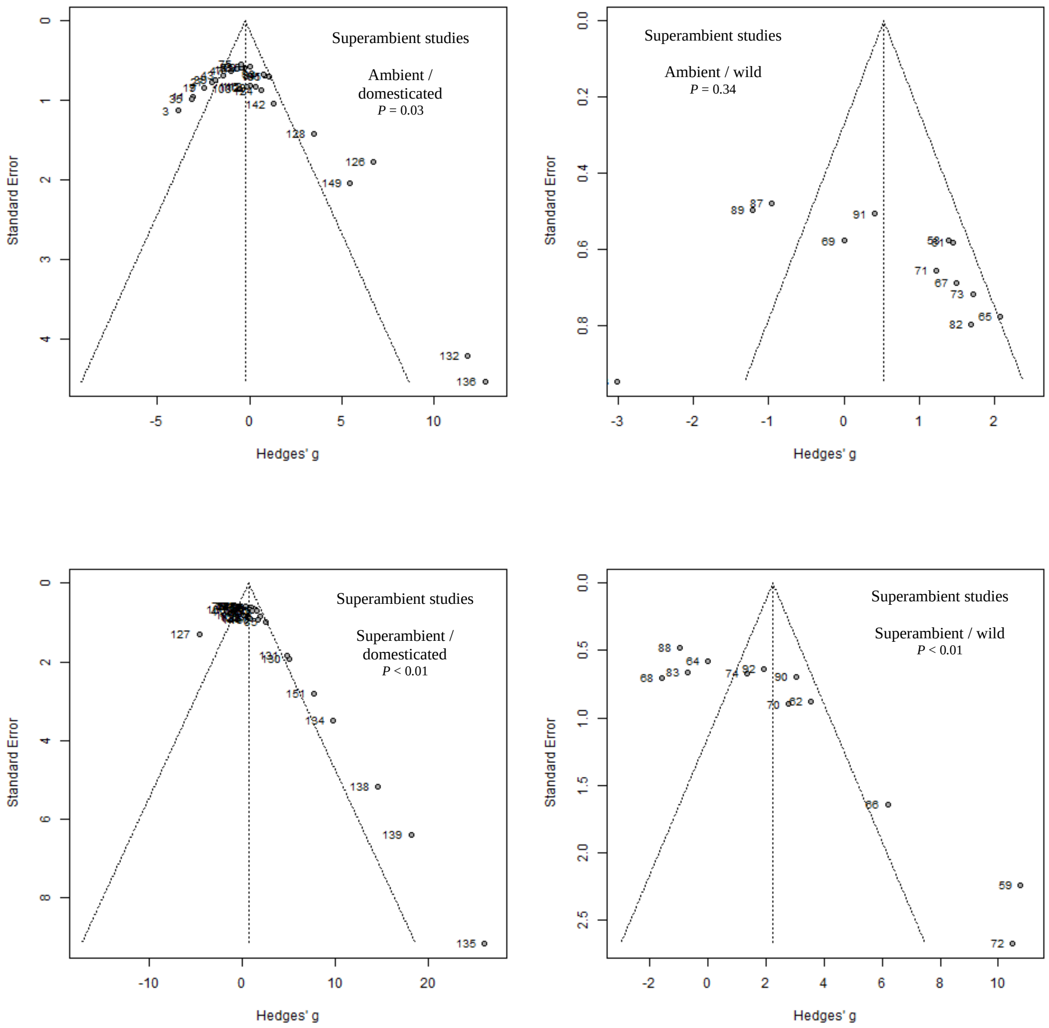
Superambient studies
Ambient / domesticated
P = 0.03
Superambient studies
Ambient / wild
P = 0.34
Superambient studies
Superambient / domesticated
P < 0.01
Superambient studies
Superambient / wild
P < 0.01
Standard Error
Hedges' g

Phosphorus

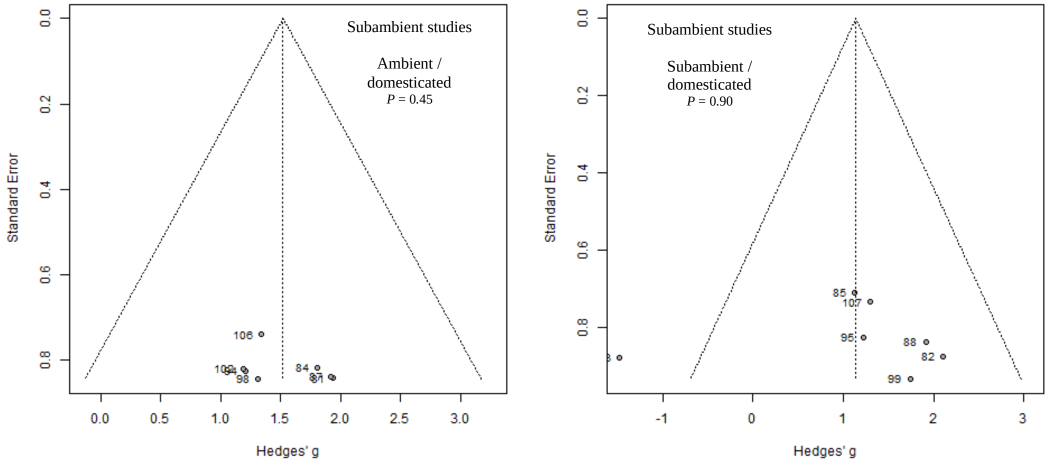
Subambient studies
Ambient / domesticated
P = 0.45
Subambient studies
Subambient / domesticated
P = 0.90
Standard Error
Hedges' g

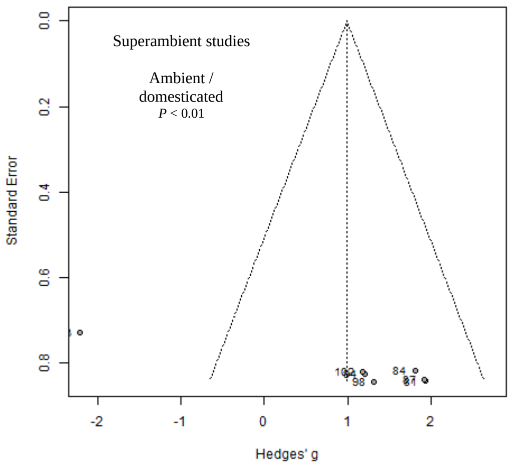

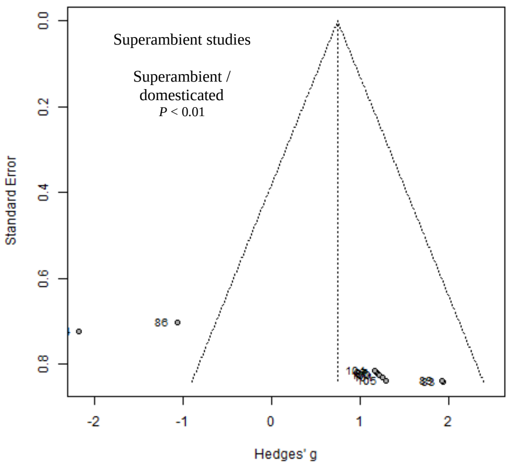

Colonization

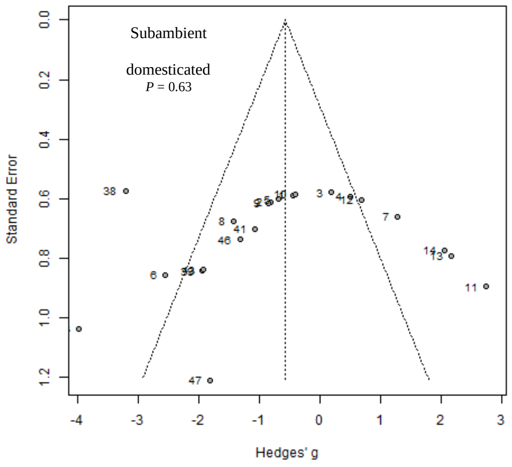

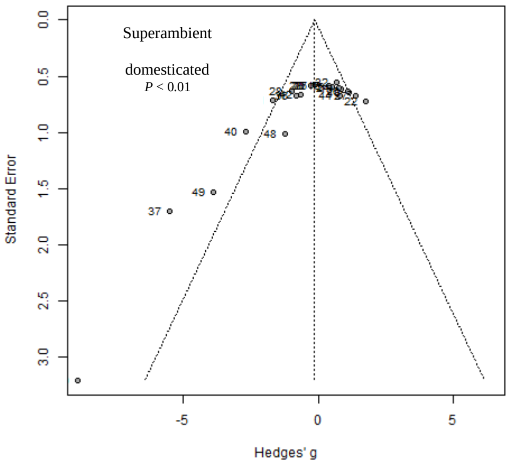

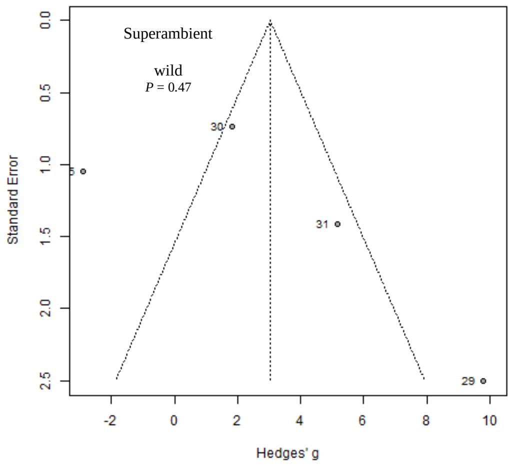

CO_2

Biomass

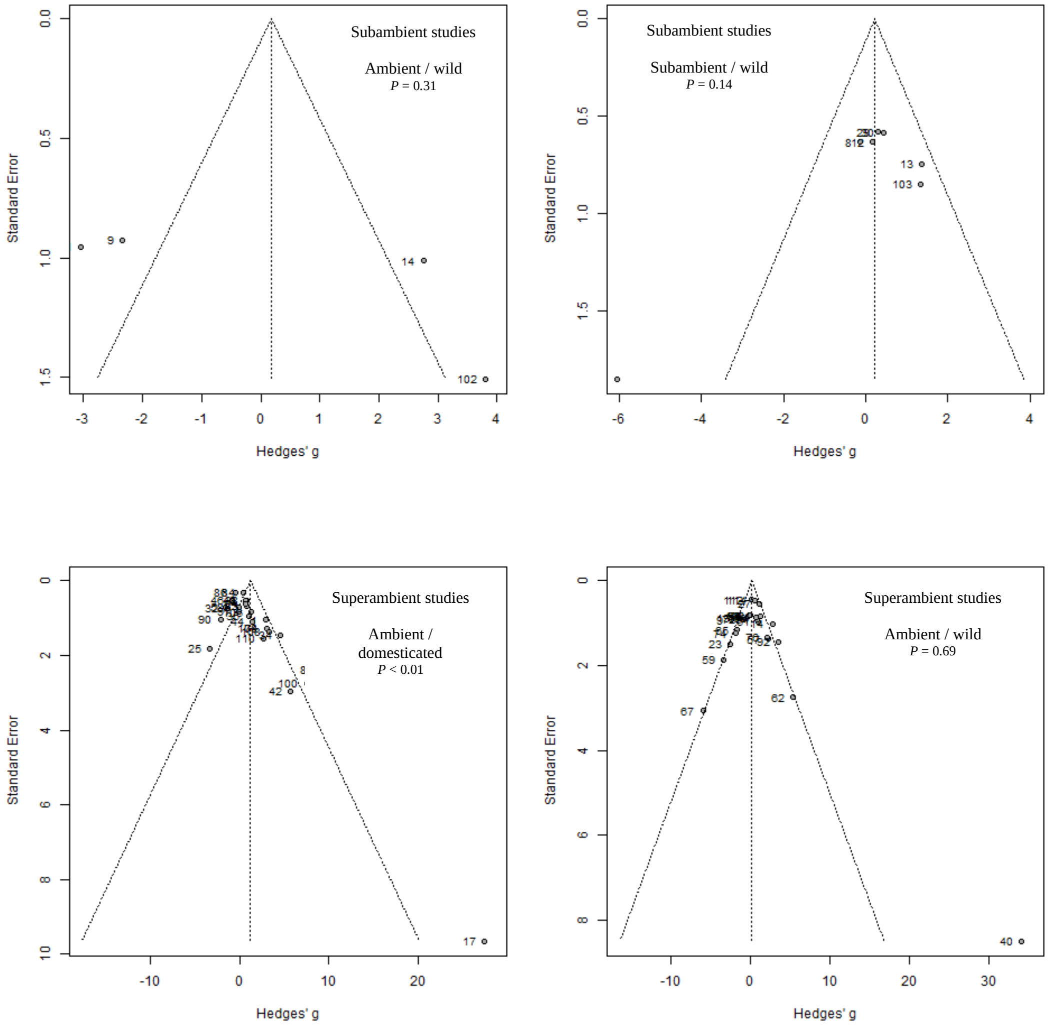
Subambient studies
Ambient / wild
P = 0.31
Standard Error
Hedges' g
Subambient studies
Subambient / wild
P = 0.14
Standard Error
Hedges' g
Superambient studies
Ambient / domesticated
P < 0.01
Standard Error
Hedges' g
Superambient studies
Ambient / wild
P = 0.69
Standard Error
Hedges' g

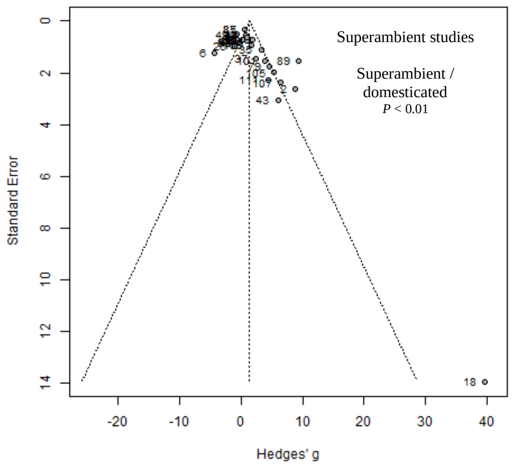
Superambient studies
Superambient / domesticated
P < 0.01
Standard Error
Hedges' g

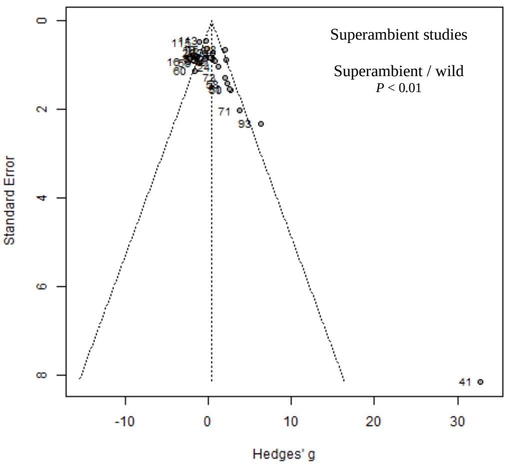
Superambient studies
Superambient / wild
P < 0.01
Standard Error
Hedges' g

Phosphorus

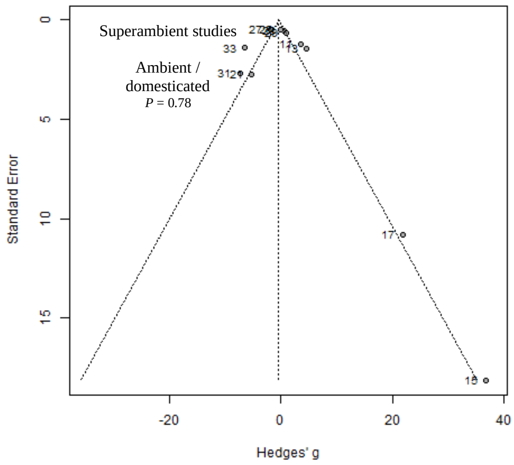
Superambient studies
Ambient / domesticated
P = 0.78
Standard Error
Hedges' g

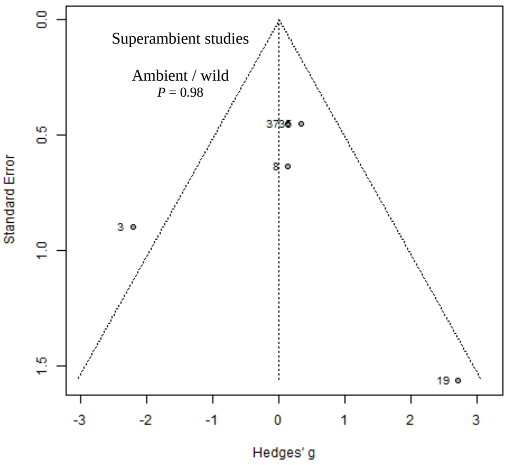
Superambient studies
Ambient / wild
P = 0.98
Standard Error
Hedges' g

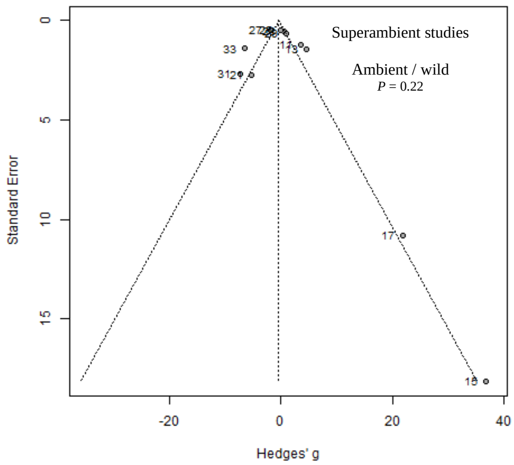
Superambient studies
Ambient / wild
P = 0.22
Standard Error
Hedges' g

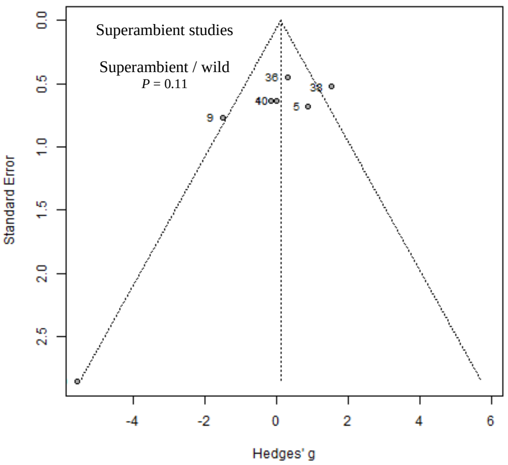
Superambient studies
Superambient / wild
P = 0.11
Standard Error
Hedges' g

Nitrogen

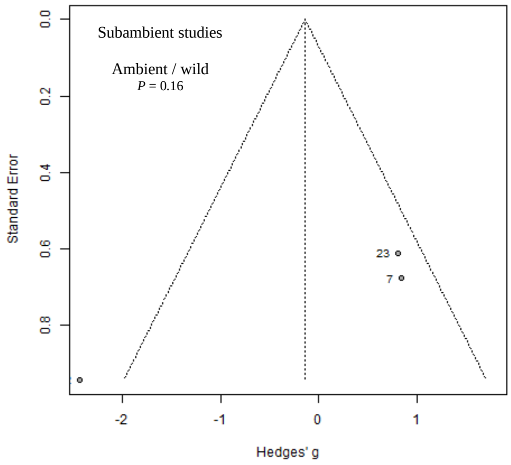

Subambient studies
Ambient / wild
P = 0.16
Standard Error
Hedges' g

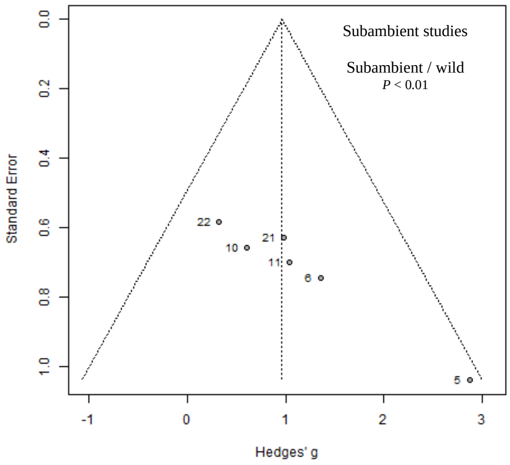

Subambient studies
Subambient / wild
P < 0.01
Standard Error
Hedges' g

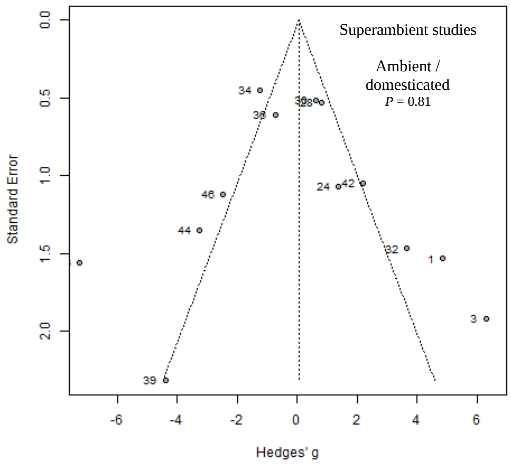

Superambient studies
Ambient / domesticated
P = 0.81
Standard Error
Hedges' g

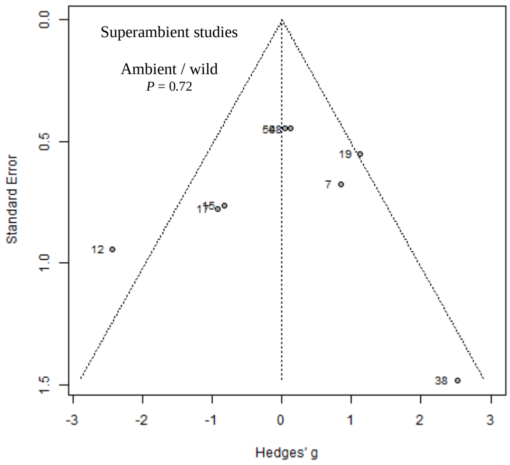

Superambient studies
Ambient / wild
P = 0.72
Standard Error
Hedges' g

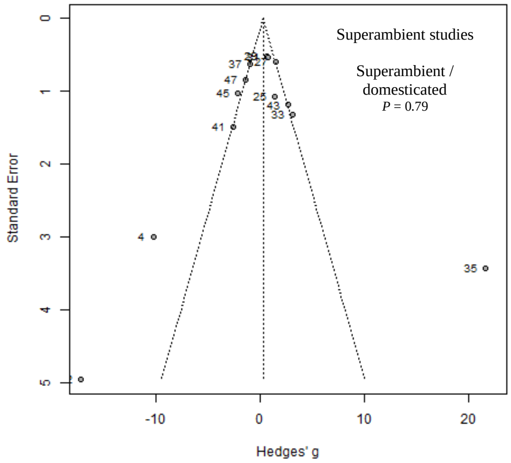

Superambient studies
Superambient / domesticated
P = 0.79
Standard Error
Hedges' g

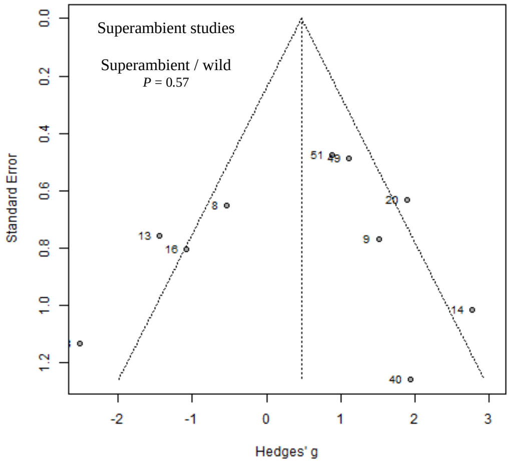

Superambient studies
Superambient / wild
P = 0.57
Standard Error
Hedges' g

Colonization

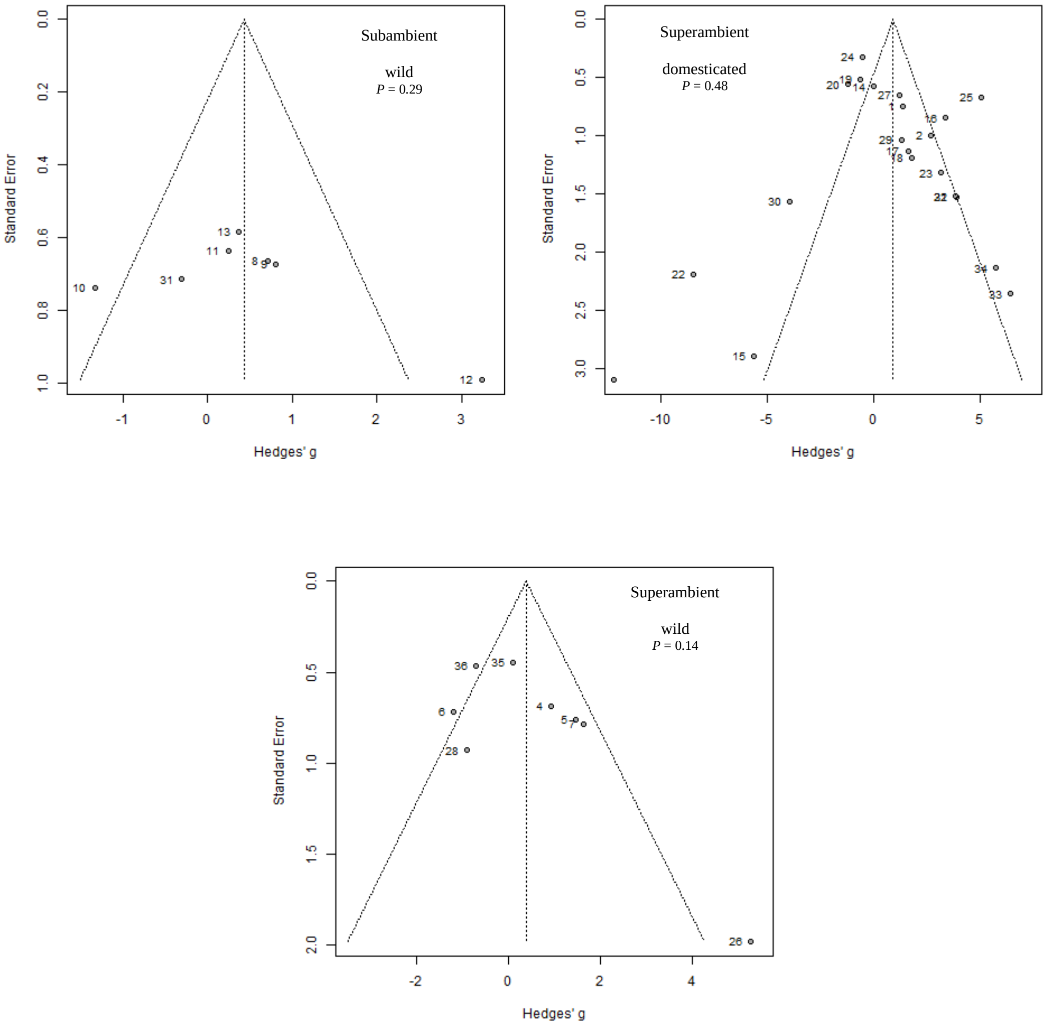

Supplement: Supplementary file 2 — Fig S2 [file ECE3-12-e8518-s003.pdf]
